# Implementing nursing diagnoses and care for the spiritual dimension of people with cancer: educational actions

**DOI:** 10.1590/1980-220X-REEUSP-2023-0141en

**Published:** 2023-12-04

**Authors:** Raniele Araújo de Freitas, Tânia Maria de Oliva Menezes, Gilberto Tadeu Reis da Silva, Raúl Fernando Guerrero-Castañeda, Halanna Carneiro Guimarães Bastos Moura, Emanuela Santos Oliveira, Larissa Simões da Cruz Pessoa, Jéssica Santos Costa

**Affiliations:** 1 Universidade Federal da Bahia, Escola de Enfermagem, Salvador, BA, Brazil.; 2 Universidad de Guanajuado, Campus Celaya-Salvatierra: Celaya, Guanajuato, México.

**Keywords:** Spirituality, Nursing Care, Nursing Process, Learning, Neoplasms, Espiritualidad, Atención de Enfermería, Proceso de Enfermería, Aprendizaje, Neoplasias, Espiritualidade, Cuidados de Enfermagem, Processo de Enfermagem, Aprendizagem, Neoplasias

## Abstract

**Objective:**

To establish the implementation of nursing diagnoses and care for the spiritual dimension of people with cancer.

**Method:**

Action research in a university hospital in the north-east of Brazil. Nine nurses and thirteen nursing technicians from the Onco-hematology and Bone Marrow Transplant Unit of this hospital took part. Data collection took place in four phases and involved the talking map technique, pedagogical workshops and a logbook. The groups’ speeches were coded using Maxqda software, subjected to Braun and Clarke’s thematic analysis and interpreted in the light of Paulo Freire’s constructs.

**Results:**

Phase 1 sought to apprehend the participants’ prior knowledge on the subject; in phase 2, proposals emerged for spiritual care organized in the Nursing Process; in phase 3, the diagnoses and care plan for the spiritual dimension for clinical practice were contemplated; and in phase 4, through the final evaluation, it was possible to see the transformations that occurred in the nursing team’s practice with the proposed implementation.

**Conclusion:**

The educational actions provided significant learning for the nursing team and the implementation of diagnoses and nursing care for the spiritual dimension of people with cancer.

## RESUMO

**Objetivo:**

Estabelecer a implementação de diagnósticos e cuidados de enfermagem à dimensão espiritual da pessoa com câncer.

**Método:**

Pesquisa-ação, em um hospital universitário do Nordeste do Brasil. Participaram nove enfermeiros e treze técnicos de enfermagem da Unidade de Oncohematologia e Transplante de Medula Óssea do referido hospital. A coleta de dados aconteceu em quatro fases e envolveu a técnica do mapa falante, oficinas pedagógicas e o diário de bordo. As falas dos grupos foram codificadas com auxílio do software Maxqda, submetidas à análise temática de Braun e Clarke e interpretadas à luz dos construtos de Paulo Freire.

**Resultados:**

A fase 1 buscou apreender o saber prévio dos participantes sobre a temática; na fase 2, surgiram propostas para o cuidado espiritual organizado no Processo de Enfermagem; na fase 3, contemplou-se os diagnósticos e plano de cuidados à dimensão espiritual para a prática clínica; e na fase 4, através da avaliação final, foi possível constatar as transformações ocorridas na prática da equipe de enfermagem com a implementação proposta.

**Conclusão:**

As ações educativas proporcionaram a aprendizagem significativa da equipe de enfermagem e implementação dos diagnósticos e cuidados de enfermagem à dimensão espiritual da pessoa com câncer.

## RESUMEN

**Objetivo:**

Establecer la implementación de diagnósticos y cuidados de enfermería para la dimensión espiritual de personas con cáncer.

**Método:**

Investigación-acción en un hospital universitario del nordeste de Brasil. Participaron nueve enfermeros y trece técnicos de enfermería de la Unidad de Oncohematología y Trasplante de Médula Ósea de este hospital. La recogida de datos se realizó en cuatro fases e incluyó la técnica del mapa parlante, talleres pedagógicos y un cuaderno de bitácora. Los discursos de los grupos se codificaron con el programa Maxqda, se sometieron al análisis temático de Braun y Clarke y se interpretaron a la luz de los constructos de Paulo Freire.

**Resultados:**

La fase 1 buscó captar los conocimientos previos de los participantes sobre el tema; en la fase 2, surgieron propuestas de cuidados espirituales organizados en el Proceso de Enfermería; en la fase 3, se contemplaron los diagnósticos y el plan de cuidados de la dimensión espiritual para la práctica clínica; y en la fase 4, a través de la evaluación final, fue posible ver las transformaciones ocurridas en la práctica del equipo de enfermería con la implementación propuesta.

**Conclusión:**

Las acciones educativas proporcionaron aprendizajes significativos para el equipo de enfermería y la implementación de diagnósticos y cuidados de enfermería para la dimensión espiritual de las personas con cáncer.

## INTRODUCTION

Establishing care based on the stages of the Nursing Process (NP) enables coherent judgment and appropriate decision-making, with nursing interventions based on a scientific method, which provides humane, ethical and effective professional action. The NP also allows for the systematic reassessment of the results obtained with the use of care interventions, as well as the reprogramming of previously planned actions^(1)^.

In the real world, it can be seen that nursing care is still based on a Cartesian, fragmented biologicist logic, with a model that prioritizes attention to the physical dimension, to the detriment of caring for the connections that make up the whole of the person’s existence, including the spiritual. On the other hand, nurses have realized that spirituality has an impact on physical health, working to improve quality of life and provide greater survival and shorter hospital stays. However, there are still difficulties and those difficulties still jeopardize the holistic care so often mentioned in theory^(2)^.

It is well-known that throughout life, people are stimulated at various times to seek balance, well-being and copingstrategies: on the one hand, there are demands from interpersonal relationships with the environment in which they live and, on the other, the need to maintain physical, mental, social and, why not, spiritual well-being^(3)^. In this context, there is the involvement of chronic diseases, cancer being a disease that implies a situation of significant stress, from the diagnosis phase to the most advanced stages, since it triggers ideas of suffering, death and finitude^(4)^.

There are evidences of associations between resilience, suffering and spirituality, concluding that the spiritual dimension of people with cancer should be included in health interventions, since it seems to be related to greater existential growth, adaptation to the disease, functional well-being and quality of life^(5)^. In this study, any mention of the spiritual dimension refers to spirituality and religiosity.

In order to understand spiritual care, it is necessary to understand the concepts of spirituality and religiosity. Spirituality refers to the intimate dimension of the person, their connection with the sacred and the transcendent, while religiosity is one of the ways of expressing spirituality, referring to organized systems of beliefs, rituals and religious practices with which the person identifies and connects to a higher being^(6)^. Thus, spiritual care involves promoting connection with others and the community, inquiring about spiritual needs and religious beliefs. In the context of illness, spiritual needs can be expressed in last decisions, reflection on the meaning of life, plans for the future, anxiety, denial, loneliness, requests for emotional support, family relationships, religious needs and minimizing suffering^(7)^.

Healthcare is not reduced to biological aspects, and professionals should include diagnoses and interventions relating to the religious and spiritual dimensions throughout the care process, thus favoring health outcomes. Spiritual care must therefore be a structured process, centered on the patient and taking into account their understanding of being and existing in the world^(8)^. Health professionals must be able to identify these demands in order to systematize care for spiritual and religious needs^(9)^.

It is known that nurses must assess the spiritual needs and promote the spiritual health of patients. However, when exploring the experience and perception of these professionals about spiritual care, a study^(10)^ showed that some nurses’ care plan was hierarchized according to the patient’s needs, with priority given to those of a physiological nature. Despite the importance given to the patient’s spiritual dimension, and the responsibility of nurses in meeting those needs, this care, whenever it takes place in clinical practice, is usually unsystematic and associated with religious manifestations.

For the purposes of NP, nurses use the taxonomic classification system II proposed by the North American Nursing Diagnosis Association (NANDA), which is a guideline for care, citing, among many Nursing Diagnoses (ND), impaired religiosity, disposition to improved religiosity, risk of impaired religiosity, risk of spiritual suffering and spiritual suffering^(11)^. However, these aspects are often not used, nor is the respective nursing care, even though they are provided for and described in the Nursing Outcomes Classification (NOC) and the Nursing Interventions Classification (NIC).

Against that background, the research question that guides this study is: Do educational actions with the nursing team contribute to the implementation of nursing diagnoses and care aimed at the spiritual dimension of people living with cancer? The aim of the study was to participatively implement nursing diagnoses and care aimed at the spiritual dimension of people living with cancer. The object of the study is: It is believed that, through educational actions with nursing professionals in the oncology service, it is possible to promote processes of co-participatory reflections on clinical practice, in order to strengthen the understanding of the importance of assistance to the spiritual dimension for the chronically ill person and/or to stimulate spiritual and religious practices, not only when the person under care expresses this demand. Such care is relevant because it brings the whole person into the context of nursing care, and the lack of care for the spiritual dimension leaves holistic care fragmented, focusing only on the disease, and does not consider the possibility that the person with cancer can benefit from the stimulation/development of their spirituality and religiosity in their illness process.

After structuring the phenomenon into codes and themes, it was possible to understand the educational actions of the action-research and the essence of the data with Freirean constructs^(12)^, since the theoretical anchoring in this author deals much more with a social and philosophical perspective than a merely methodological one. In this framework, there is an ethical and existential component behind concepts such as dialog, limit situations, emancipation and liberation.

In addition, the aim is to expand new knowledge about this incipient subject in the scientific literature, as well as to revive attention to the spiritual dimension in nursing, which has been forgotten over the years. For this reason, it can be seen that, in most health situations, this dimension is excluded from the NP, so that care, in practice, does not encompass the biopsychosocial-spiritual being.

## METHOD

### Study Design

This is a qualitative study based upon the methodological framework of action-research. Action-research is defined as a research method or strategy that brings together various social research techniques, with which a collective, participatory and active structure is established at the level of capturing information^(13)^.

The recommendations of the Consolidated Criteria for Reporting Qualitative Research^(14)^ guide were followed for this study.

### Location

The research setting was the Onco-hematology and Bone Marrow Transplant Unit of a university hospital in northeastern Brazil.

### Population and Selection Criteria

Nine nurses and 13 nursing technicians working in the aforementioned hospital unit participated in this study. Nurses and nursing technicians who had been working in the unit for at least six months, which was considered sufficient time for them to become familiar with caring for people with onco-hematological diseases, and who belonged to the unit’s permanent staff, agreed to take part in the group activities on a voluntary basis. Exclusion criteria were: nurses and nursing technicians who were on vacation, maternity leave or medical leave and professionals who were not permanently employed at the unit. Two nursing technicians who performed exclusively administrative activities were excluded.

### Sample Definition

The criterion of saturation of information was used, therefore, data collection was terminated when the participants’ statement meanings began to show regularity^(15)^. Furthermore, in the action-research strategy, when the number of participants is very large, the possibility of using samples or representativeness of the research population is considered^(13)^.

### Data Collection

Data was collected between August 2022 and February 2023 using individual questionnaires, a field diary and two group collection techniques (talking maps and educational workshops).

The interviews were carried out by the main researcher and a nursing student, on pre-scheduled dates and times, ensuring that the environment was private and provided privacy for the participants. The action-research followed the following phases:

1^st^ phase: Situational diagnosis, composing the talking map in three moments. Initially, a questionnaire was administered to characterize the participants, as well as questions about concepts of spirituality, religion and religiosity, in order to understand how they understood care for the spiritual dimension in the context of cancer. In the second approach, the difficulties and facilities in inserting spiritual care for people with cancer were discussed. In the subsequent approach, the discussion moved on to the intervening factors in the use of NANDA-II diagnoses in the NP, as well as spiritual care for the possible problems generally identified in people with cancer.

2^nd^ phase: Planning educational actions with the group. Based on the problems shared and the topics that generated discussion, the educational activities were planned through three workshops. Planning consisted of defining the actions that would contribute to solving/equalizing the problems detected, as well as the objectives of these actions, the means needed to achieve them and the people who would carry them out^(13)^. It was necessary to address Wanda Horta’s theory^(16)^ and NANDA Taxonomy II, as they are, respectively, the nursing theory and taxonomy adopted in the site’s nursing process.

3^rd^ phase: Implementation of the actions – educational workshops. The implementation of the actions followed a script for each workshop. At the end of each workshop, partial analyses and evaluations were carried out in order to readjust the strategies for continuing the action research. This movement is understood as the cycle of action-research, going from “action-reflection” to “learning-action”^(13)^. At the end of the workshops, the NANDA-II diagnoses and nursing care for the spiritual dimension were implemented in practice, included in the NP and in the hospital’s electronic medical records. Thus, at the end of this phase, the researcher provided the diagnoses and nursing care, constructed collectively in the educational workshops, which were added to the computerized system.

4th phase: Final evaluation of the action-research process- this took place through the researcher’s participant observation, through visits to the unit, observing the electronic medical records, nursing prescriptions and through on-site dialog with the nursing team. After three months, a final workshop was held with the aim of evaluating, together with the nursing team, their participation in the study and its repercussions on their working reality. In this session, the knowledge built up by problematizing the group, the experience with the educational action applied and the learning in the research process were discussed.

Each group activity lasted approximately three hours. The content was recorded in the field diary and by audio recording, using a device for this purpose, with the consent of the participants. The speeches were then transcribed in full.

### Data Analysis and Processing

The data was imported, transcribed and coded using MAXQDA plus 2020 software^(17)^. Data analysis followed the six phases of Braun and Clarke’s thematic analysis^(18)^, and the results of the educational actions were interpreted in the light of Paulo Freire’s constructs^(12)^.

### Ethical Aspects

The study complied with all the guidelines and legal prerogatives established by Resolutions 466/2012 and 510/2016 of the National Health Council. The matrix project was cleared by the Research Ethics Committee under Opinion No. 5.530.277/2022. All participants received guidance and explanations about the study, as well as the Informed Consent Form (ICF), which was duly signed after agreeing to take part in the research. Participants were guaranteed confidentiality and anonymity by using the codenames “E” for nurse and “TE” for nursing technician, followed by a numeral indicating the sequence of speech.

## RESULTS

A total of 22 nursing professionals participated in the study. With regard to professional category, 13 (59.1%) were nursing technicians and nine (40.9%) nurses. Ages ranged from 29 to 60, with a mean of 38. Thirteen (59.1%) participants declared themselves brown, six (27.3%) black and three (13.6%) white. Sixteen (72.7%) participants were females and six (27.3%) males. Regarding the highest degree obtained, eight (36.4%) had a specialization in oncology (all nurses); one (4.5%) nurse had a doctorate; and seven (32%) nursing technicians had a degree (biology or nursing).

With regard to the length of time they had worked in nursing, there was a variation between six and 23 years; in oncology, the time varied between six months and 18 years. The majority (82%) had one job and four (18%) had two jobs, ranging from 36 (in one job) to 72 hours a week. In terms of religion or belief, ten (45.4%) were Catholics; six (27.3%) Evangelicals; one (4.5%) Jehovah’s Witness; one (4.5%) Spiritism; and four (18.3%) had no religious affiliation, one of whom declared himself an atheist.

Data production involved participants and the researcher constructing actions that could culminate in the implementation of diagnoses and nursing care. To do this, we used activity techniques for collective construction, namely: a talking map for diagnosing prior knowledge and programming actions and educational workshops containing themes to be worked on.

The processing was based on the lexicons identified as significant in the corpus of speeches transcribed during the research phases, from which the codes were extracted, organized and put into themes by means of thematic analysis. In the trajectory of revolutionary transformation of the nursing care provided, the educational actions made it possible to identify the following themes: a) Awareness of non-systematized spiritual care in the NP; b) Collective planning of activities and implementation of actions in search of meaningful transformation of the reality experienced; and c) The effectiveness of actions in meaningful learning and in the proposed implementation with the NP.

a) Awareness of unconscious and unsystematized spiritual care in the Nursing Process.

This theme emerged in the first phase of the action research, the situational diagnosis phase, using the talking map data collection technique. It was necessary to identify the participants’ prior knowledge about ND and nursing care for the spiritual dimension of people with cancer, and such diagnoses were possible in the first activity of the talking map: There are different concepts about spirituality, religiosity and faith; Although they are connected, it is possible to recognize the difference between spirituality and religiosity; Spirituality is something related to belief, hope, and makes the person with cancer choose to continue in the daily struggle. Religiosity is the practice of religion, linked to beliefs, temples and religious objects.

The participants mentioned that the spiritual dimension includes spirituality and religiosity. Defining religiosity is easier than defining spirituality, so spiritual care becomes more accessible to nursing professionals and religious people, as they always have objects related to the Sacred in the unit’s beds.

In the second activity, the participants pointed out the following topics: The dimension of spirituality and religiosity of people with cancer is important because it provides strength, comfort and gives new meaning to daily life with cancer, improving coping; All patients should receive care for the spiritual dimension, including those who have no religion or beliefs (atheists and agnostics); It is very likely that all patients have unidentified demands of a spiritual/religious nature; It is important to work on the spiritual dimension of people with cancer, but professionals don’t know where to start with this approach.

In the third activity with drawings, conversations and connections, the participants came to the following conclusions: There are facilities and difficulties in offering care to the spiritual dimension of the person with cancer; There is no known and clear care about nursing problems, or possible problems in this context; It is necessary to respect people’s beliefs, since the fear of being invasive when talking about religion exists among professionals; The nursing team identifies this unconscious care, as they encourage the hospitalized person to cling to God, when they know about their religion; The team does not recognize this care in the nursing prescription; Although there is no specific care for the spiritual dimension in the NP, the team talks to the person with cancer about faith, hope, trust and positive thoughts.

The speeches below report the problematization about the spiritual care necessary for comprehensive care for people living with cancer:

*(...) to tell you that I assess this part when I make the nursing diagnosis... I don’t. But I do know that stimulating spirituality and religiosity has benefits for them. Positive thoughts, a reduction in anxiety and depression and, whether they like it or not, these benefits are reflected in their physical state. So I think it’s worth working on this aspect (E1).*

*Knowing about it, I would have been prepared. Spirituality is an issue that can be incorporated into our unit’s nursing process, which is something relevant that needs to be considered and schematized in our work. We talk about it, but nothing is written down (...) because we lack preparation. But in the end, as we discussed it more, we came to the conclusion that it’s about what we already do a bit in the service and we can improve a lot (E4).*

*It will be very good to have this here, because we have situations in which patients say they are depressed, or afraid, anxious, suicidal (TE11).*

E1 comments that he doesn’t use nursing diagnoses, which may infer that he doesn’t identify spiritual problems either, but he understands that spirituality/religiosity play a role as a resilience strategy that can be used by people suffering from cancer. For him, it is necessary to work with the study proposal. E4 reports that by knowing how to implement the steps for systematized spiritual care in PE in practice, what is already being done in the unit can be improved and incorporated as nursing care. In addition, TE11 highlights a working practice in which patients express fear, depression and suicidal thoughts, which is why he believes that the care in question is beneficial for these people.

b) The collective planning of activities and the implementation of actions in search of a significant transformation of the reality experienced.

After the situational diagnosis phase, the researcher and the participants had to work together to create a strategic plan with actions. In the planning phase, the following question was posed: what can be planned and done to ensure that care for the spiritual dimension of people with cancer takes place in the routine of nursing professionals? After the debate and group construction, it became clear that it was necessary to work from the nursing history to the evolution, including the notes and the shift handover, in order to make the practice of systematized spiritual care a routine in nursing care.

The Chart 1 below describes the actions planned and validated by the participants, which made the educational workshops possible, resulting from the search for a change in reality based on the participants’ action-reflection and critical awareness:

**Chart 1:** Activities planned for the implementation phase of the action research – Salvador, Bahia, Brazil, 2022.

PROPOSED ACTIVITY	TOPICS ADDRESSED FOR THE PROPOSED IMPLEMENTATION	PARTICIPANTS
– Educational workshop 1	– The Nursing Process – Wanda Horta’s theory and spiritual needs – Nursing history and nursing problems related to spirituality and religiosity	– Nursing team
– Educational workshop 2	– Nursing Diagnoses – NANDA-II – addressing domains of spirituality, religiosity, meaning of life and hope – Expected results on spiritual and religious needs – NOC	– Nurses
– Educational workshop 3	– Nursing care about the spiritual dimension and Nursing Prescription containing this care – NIC – Nursing evolution and notes in this context	– Nursing team

The implementation phase took place through three educational workshops. A fictitious case study was created by the researcher, containing nursing problems related to spirituality and religiosity; this case study was used in the three workshops, and the participants were able to make practical simulations and selections for the spiritual anamnesis, nursing diagnoses and nursing care related to the spiritual dimension that were feasible in their practices. For the workshops, the nursing history adopted by the hospital was used, with new proposals for questions on the spiritual dimension and the NANDA, NOC and NIC books. In the workshop on NANDA-II nursing diagnoses and the NOC, only nurses took part, as they are contained in the EP phases and logical reasoning is the preserve of this professional category. The other topics described in the table were discussed with nurses and nursing technicians.

The following statements reflect the collective construction of the workshops through problematization:

*It was important to raise the problems of nursing, which is not given much attention to this spiritual part, is showing how to do and being relevant (TE3)!*

*I realized that NANDA-II makes it clear that nursing care goes beyond the body, and there is this section on oncology care, and there is spiritual support there, as there is in the NOC. These diagnoses focused on spirituality and religiosity will be key pieces here. (...) We’re really surprised by how well NANDA-II and NOC have worked. Now let’s give importance to what is also important (E2).*

*For me, reflecting with the group on care focused on spirituality and religiosity was the most important point of all the workshops. It was reconnecting with what can be used by the nurse (...) I felt reconnected with something I had lost. We deal with everything that is most human in us every day. No profession does that!” (E8).*

TE3 reports on the importance of understanding which patients’ spiritual demands merit intervention. E2 was surprised to discover the diagnoses and expected results relating to the subject of the study, showing that there is scientific backing for planning care for the spirituality and religiosity of the person under nursing care. E8 emphasizes the importance of reflecting as a group when listing feasible care for the unit, as well as bringing it closer to an action that is the responsibility of nursing.

At the end of the implementation phase, the diagnoses and nursing care listed were made available to the person responsible for the hospital’s NP, and were parameterized in the electronic medical record system for use in the unit.

c) The effectiveness of the actions in meaningful learning and the proposed implementation of the nursing process.

In the final workshop to evaluate the educational action to implement the proposal, it was possible to see the benefits provided through the participants’ speeches. The speeches below portray the positive impact of the educational action and critical-reflective learning:

*It was very productive, for the professional, care and personal part, especially since we are working with oncology patients, and they demand a lot about this. (...) So, the opportunity to learn more about spiritual care, we can go far, it gave us a direction (E6).*

*And so... I go straight to the history, I already see the psycho-spiritual part there (I used to fill it out very little, but after the workshops... there’s no way I won’t fill it out more), then I remember the diagnoses, I remember the care... That was good, one thing is already signaling the other (E9).*

*It was so good to take part in the workshops (...) we’re always using the records here and remembering to take this into account with the patients. Today I take a different approach (E10).*

*We used to do something about spiritual care, but we didn’t consider it care. Today we do and we’re aware of it. We managed to understand that spiritual care goes hand in hand with biological care and, since it’s in the prescription, we incorporated it as care (TE6).*

The participants reported experiences in offering care to the spiritual dimension, which until then had never been practiced. The speech below describes the awakening to action, after action-reflection-action and problematization in educational activities:

*Can you believe I said a prayer with a patient twice? I’d never done that, it gave me a thrill, and I felt it did them so much good. It was like this: one patient was after a bone marrow transplant and the other was in palliative care, the latter was very weak, not talking, I asked him if he was practicing his prayers, he said no, he was lucid and oriented, so I invited us to say a prayer together... I held his hands, and even his family member asked to take part. At the end of the next shift he passed away, and I felt different for having done this with him. It was incredible, because I’d never done it before or even thought of doing it (E6).*

*Myself, when I get to the bedside and see that people are praying, I leave and come back later. If it’s not urgent, I let the patient practice their religiosity... I reflected a lot on respect in the workshops, and I see that it makes a difference for them, because the journey is grueling. And I need to encourage their moment of faith (TE6).*

E6’s and TE6’s statements show that the nursing team is becoming more aware of the spiritual dimension of care and the effectiveness of the educational action, highlighting practices that until that moment, had not been seen or done in the unit.

## DISCUSSION

Regarding the study’s proposal to carry out an educational action to implement nursing diagnoses and care for the spiritual dimension of people with cancer, it is possible to analyze nursing care from a Freirean perspective, in order to understand the act of caring/educating. Thus, it converges with the assumption that education is not neutral and that the care process is educational and a political act^(13)^, which can be taken on by the nursing team as a transformative proposal.

The research was carried out using the action-research methodology, as a research approach in which collective participation is the principle that defines the entire research process^(19)^. The theory understands education as an encounter between human beings, mediated by the world. For the dialogic, problematizing educator-learner, the programmatic content of education is not a donation or imposition, that is, a set of information to be deposited in the students, but the organized and systematized return of what was given to them in an unstructured way^(12)^.

The theorist advocates transformation through problematizing education, which proposes mobilizing the construction of knowledge based on meaningful experiences. Meaningful learning seeks to build an educational process based on students’ previous knowledge, with the aim of making the educational process more dynamic. There is a continuous need to transform pedagogical practice into a moment of pleasure and satisfaction for both the educator and the student, in order to make the educational process more effective^(13)^.

The transformation that took place in this study through group activities, using the talking map technique and educational workshops, since group work can be a facilitating strategy in the teaching and learning process, as it enables participants to share knowledge. Mutual help makes it possible to learn from others. Group activities break down the vertical learning relationship that exists between the educator and the student, allowing for a horizontal relationship that favors building on the group’s intentions^(20)^. The use of active technologies is an educational strategy for group work, with the use of methods and dynamics such as games, workshops and focus groups, which value listening and the interest of those involved, considered low cost and more accessible, and characterized as light technologies that can be easily applied upon acceptance by the group^(21,22)^. In this study, soft technologies and active methods were essential for involving the group, overcoming shyness, as well as arousing motivation and engagement.

The initial phase of the research was essential for expanding critical reflection on dialogicity, creating bonds and trust between the researcher and the participants, and was important for a prior diagnosis of the participants’ knowledge about spirituality, religiosity, faith and spiritual care in the reality they experienced. At this stage, listening to the nursing team was fundamental for taking the next steps. Listening is something that goes beyond everyone’s ability to hear. Listening means permanent availability on the part of the listener to open up to the other person’s speech^(20)^. It’s by listening well that I prepare myself to better position or situate myself from the point of view of ideas.

Gathering information, based on experience and living with the study’s object of investigation, opens up space for initial dialog and planning about what is intended to be addressed or transformed, what is a priority or not, what will be feasible and what is close to the reality of the nursing team. Thus, the dialog began with an approximation of the research problem, seeking to understand it in order to carry out the situational diagnosis.

Only through dialog there is communication. And when the two poles of the dialog connect in this way, with love, with hope, with faith in each other, they become critical in the search for something. A relationship of sympathy is then established between the two. Only then is there communication. Through the dialog established in the initial phase of the research, it was noted that the participants were open to the new, which makes it possible to experience transformation. For the theorist, true education is only possible through dialogue, and an educational program should not be carried out by just one of the interested parties^(23)^.

The participants concluded that the spiritual dimension can benefit the person suffering from cancer, and argued that religion and spirituality are particularly important for people with this diagnosis. The role of the binomial, in this case, may be associated with issues such as resilience, hope and the spiritual well-being of patients, since, when experiencing the suffering caused by the disease, there is a greater connection with religiosity and spirituality, favoring high scores of religious and spiritual coping^(24)^. Freire’s theory makes use of a liberating transformation, when it places itself as a pedagogical category capable of educating for freedom, through humanization that values justice, freedom and hope, even in the face of the contradictions of the human existential condition. For the thinker, faith is the manifesto of the divine plan and therefore directs us towards solutions that are inherent to human beings^(25)^.

The mediations, inferences and expositions made by the researcher sought reflection-action in a dialogical way, using light technology, envisioning that the authenticity of dialog facilitates interpersonal relationships, making the subjects feel valued and aware of the importance of their social role and the transformation of reality^(26)^.

The participants reflected that care for the spiritual dimension took place in an informal and unsystematized way. This problematizing awareness is aimed at achieving the greater purpose of education, which is to prepare human beings to become aware of their world and to act intentionally to transform it, always for the better, towards a world and a society that allow a more dignified life for man himself, in this case, the person with cancer^(10)^. In other words, in the practice of conscientization, the participants initially made a spontaneous approach (naive position) to their concrete situation (reality). By overcoming this, they came to experience, apprehend and unveil this reality, through a critical development of awareness, penetrating the phenomenon and analyzing it^(27)^.

At the end of the diagnostic phase, we moved on to shared diagnosis and action planning, in which we were able to awaken together (researcher and participants) to systematized care for the spiritual dimension, incorporated into the SP. The speeches about the need to improve the nursing history, the implementation of the NANDA-II diagnoses and nursing care in the context of the spiritual dimension of people with cancer showed what Freire an theory^(20)^ says about man’s consciousness and his unfinished state, which has an ontological vocation to become more. This characteristic is inherent to the individual, it is part of their cultural, historical and action function in relation to nature. The being learns and teaches with others, with nature, with the Creator, in other words, it is a social being that has a disposition for integration.

Themes were proposed to be worked on with the participation of those involved, in order to stimulate strategic and problem-solving capacity in a relationship of solidarity, as well as awareness and recognition of an uncovered experience that needs intervention, in order to improve the care offered to people with cancer. This exercise was only possible because it encouraged people to be aware of and transform reality through their creativity, shared knowledge and dialog, in which the word became the praxis for transforming reality. To make this dream possible, it was necessary to promote an education that stimulates awareness, which is not just knowledge or recognition, but the option, decision and genuine commitment to this process^(28)^.

During the implementation phase, the workshops covered spiritual anamnesis, nursing records, conscientious completion of the nursing history, dis(knowledge) and applicability of NDs and care to support and stimulate the spirituality/religiosity binomial of people with cancer. As the workshops took place, new ideas, suggestions, knowledge and skills were built up along the lines of liberating education, generating a list of nursing diagnoses and feasible care for the nursing prescription. It should be noted that the learning and applicability of these tools do not depend solely on the educator, but are the result of the educator-student interaction and the context in which they live^(29)^.

In order to implement the study proposal, it was necessary to move away from the banking education so criticized by Freirean theory, which uses the methodology of uncritical memorization of knowledge and contributes to the passivity of the students, so that the researcher and the participants could learn together, through dialogical problematization, to interpret the phenomenon. A dialog that respects people and their characteristics, but also provokes and challenges them to make decisions and transformations. A dialog that converges towards praxis, understood as a process of reflection, prior to action, and action committed to change. Thus, there would be no other tool than dialog as a means of delving deeper into worlds and visions^(28)^. In the search for freedom resulting from considerable experiences in education on the part of the student and educator, the theory used defends autonomy as a process of becoming, as it is something to be built, considering the dynamics of reality in all its dimensions, based on stimulating experiences^(20)^.

The evaluation phase highlighted the effectiveness of the educational actions used, in which the participants’ learning experiences provided them with diverse knowledge, enabling them to make responsible and conscious decisions about themselves, the team and the context in which they were inserted. In an activity so impregnated with the technical dimension of work, the nursing team’s care imposes challenges beyond the biologicist content. This proves the importance of transforming nursing practices, through collaboration made possible by dialogicity, in the development of autonomy that comes from revolutionary and liberating action^(12)^.

The limitation of the study is that it was carried out in just one hospital unit, but this does not exclude the opportunity this study has created for nursing teams to review their care practices for people suffering from cancer and diseases with no therapeutic possibilities, with a view to valuing spirituality/religiosity and ensuring that this care does not only include biopsychological aspects. It is worth highlighting the opportunity to promote changes in care practices based on the insights and discussions generated by the study, which may encourage other nursing teams to consider the spiritual dimension in care, in addition to the biopsychological aspects. However, further research in different contexts is needed to gain a more comprehensive understanding of the topic.

## CONCLUSION

In order to implement the proposal, value was placed on contextualizing reality in a critical and politicized way, as well as the participants’ beliefs and knowledge applied in their daily lives, having the researcher as a mediator of learning through dialogue. The researcher-nursing team relationship was enriching, open and democratic, which stimulated motivation and movement towards the desired change.

From the perspective of dialogue and problematizing-liberating education, the different forms of expression of the participants were taken into account, valuing their speeches, mediated by participatory methodologies, including the talking map and educational workshops. Respect, empathy and a horizontal relationship made it possible to recover humanization, highlighting that the humanization of subjects is undoubtedly a commitment stemming from Freirean constructs, is critically positioned and applies to various contemporary challenges, such as those presented here. By developing the educational action, the participants conquered their space in the care of the spiritual dimension and felt strengthened to continue a process in which they all believed and to which they adhered no longer as a proposal, but as a purpose of integral work, of building autonomy and liberation.

The participants in the study were able to rethink their care practice in the multidimensionality of the human being, which did not address the spiritual dimension, motivating each other to change this reality, with inclusion in the Nursing Process, which until then had not been reflected upon. In addition, in the evaluation phase of the research, it was possible to identify the impact of this approach on patients’ quality of life, feedback from participants and a desire to expand implementation to other units on the part of nursing coordination and hospital management.

The nursing team was able to act on their own, systematically advancing on a topic of common interest and they were able to act, through problematization, based on this collectively constructed knowledge. In addition, the results of this research could support nursing management by identifying strategies to implement and expand spiritual care for the sick person, as well as providing elements of the teaching-learning movement to expand spiritual care for hospitalized people, especially those experiencing cancer. It should be emphasized that the practice of participatory activities developed with nurses and nursing technicians can be successful, as it allows for open, interactive and reflective dialogue, as well as seeking the positive transformation of reality.
